# Increase in brain glycogen levels ameliorates Huntington's disease phenotype and rescues neurodegeneration in *Drosophila*

**DOI:** 10.1242/dmm.050238

**Published:** 2023-10-19

**Authors:** Akanksha Onkar, Deepashree Sheshadri, Anupama Rai, Arjit Kant Gupta, Nitin Gupta, Subramaniam Ganesh

**Affiliations:** ^1^ Department of Biological Sciences and Bioengineering, Indian Institute of Technology (IIT), Kanpur 208016, India; ^2^ Centre of Excellence in Neuroscience, Neurotechnology, and Mental Health, Gangwal School of Medical Sciences and Technology, IIT, Kanpur 208016, India; ^3^ The Mehta Family Centre for Engineering in Medicine, Indian Institute of Technology, Kanpur 208016, India

**Keywords:** Neurodegenerative disorders, Glycogen synthase, Aging, Oxidative stress, Autophagy

## Abstract

Under normal physiological conditions, the mammalian brain contains very little glycogen, most of which is stored in astrocytes. However, the aging brain and the subareas of the brain in patients with neurodegenerative disorders tend to accumulate glycogen, the cause and significance of which remain largely unexplored. Using cellular models, we have recently demonstrated a neuroprotective role for neuronal glycogen and glycogen synthase in the context of Huntington's disease. To gain insight into the role of brain glycogen in regulating proteotoxicity, we utilized a *Drosophila* model of Huntington's disease, in which glycogen synthase is either knocked down or expressed ectopically. Enhancing glycogen synthesis in the brains of flies with Huntington's disease decreased mutant Huntingtin aggregation and reduced oxidative stress by activating auto-lysosomal functions. Further, overexpression of glycogen synthase in the brain rescues photoreceptor degeneration, improves locomotor deficits and increases fitness traits in this Huntington's disease model. We, thus, provide *in vivo* evidence for the neuroprotective functions of glycogen synthase and glycogen in neurodegenerative conditions, and their role in the neuronal autophagy process.

## Teaser

**Summary:** Whereas healthy brain contains very little glycogen, aged brains and brains of people with neurodegenerative disorders tend to accumulate glycogen. We demonstrate here that, in the *Drosophila* model of Huntington's disease, enhancement of glycogen synthesis in fly brain ameliorated the disease phenotype through activation of auto-lysosomal functions.

## INTRODUCTION

Glycogen, a glucose polymer, is known for its role as a carbohydrate storage reserve within the animal kingdom (Roach et al., 2012). Most of our knowledge regarding the structure and regulation of glycogen comes from extensive studies of liver and muscle tissues (Hers, 1976; Hems and Whitton, 1980; Jensen and Richter, 2012; Hearris et al., 2018). However, with increasing evidence of abnormal glycogen buildup in the brain in diverse pathological conditions (Trivedi et al., 2003; Rohn, 2015; Augé et al., 2017; Rai et al., 2018; Riba et al., 2021a,b), there has been growing interest to understand the function of neuronal glycogen. Intriguingly, unlike liver and muscle, which store significant amounts of glycogen, the glycogen content in the brain is minimal, the majority of it primarily being stored in astrocytes (Pellerin and Magistretti, 1994; Obel et al., 2012; Saez et al., 2014). Thus, excessive accumulation of brain glycogen in pathological neurodegenerative conditions indicates the alterations in the glycogen metabolic pathways, which could either be the cause or the consequence of abnormal neuronal physiology and function (Rai and Ganesh, 2019; Onkar et al., 2020; Sheshadri et al., 2021).

A small number of emerging reports have proposed a causal role of neuronal glycogen in stress response, synaptic plasticity, aging and neurodegeneration. For example, neuronal glycogen, either as a biomolecule or as a readout of the activated glycogen synthesis machinery, has been proposed to have neuroprotective roles under physiological stress conditions (Onkar et al., 2020). Glycogen is the energy source mobilized to prevent neuronal death from hypoxia, ischemia, hypoglycemia, endoplasmic reticulum stress and oxidative stress (Hossain et al., 2014; Saez et al., 2014; López-Ramos et al., 2015). Glycogen-derived lactate in astrocytes and neuronal glycogen content are associated with memory and cognition, and glycogen depletion in the brain reduces synaptic strength, impairs memory formation and increases seizure susceptibility (Duran et al., 2019; Vezzoli et al., 2020). Increased brain glycogen, by contrast, is widely correlated to brain aging and neurodegenerative phenotypes (Rai and Ganesh, 2019). Abnormal glycogen aggregates, in the form of corpora amylacea, are commonly observed in the aging brain (Augé et al., 2017; Riba et al., 2021a,b), and ectopic glycogen build-up in healthy neurons of mouse and fly models results in accelerated aging and motor impairment (Duran et al., 2012). Recently, changes in brain glycogen levels have also been shown to modify sex-specific survival in *Drosophila melanogaster* (Sheshadri et al., 2021). Abnormal glycogen aggregates or polyglucosan bodies have also been detected in the degenerating brain of mouse models and in brains of humans diagnosed with neurodegenerative disorders (Trivedi et al., 2003; Rohn, 2015; Rai et al., 2018), although their significance is yet to be understood. A previous study from our group has demonstrated that glycogen accumulation can partially rescue the toxicity induced by mutant Huntingtin (Htt) protein in a cellular model of Huntington's disease (HD) (Rai et al., 2018). Even though this model cell line-based study advocates a novel role for glycogen in HD pathology and provides mechanistic insights into the influence that glycogen has on neuronal survival, its *in vivo* relevance has not been tested. In our present study, we show that increasing expression of glycogen synthase (officially known as *GYS1* in humans and *Glys* in *Drosophila*, but hereafter concomitantly referred to as GS) in the *Drosophila* model of HD increased lifespan and alleviates HD-related pathologies. Furthermore, overexpression of GS in the brains of HD flies aided in the reduction of mutant Htt levels as well as that of oxidative stress markers, the rescue of lysosomal defects and the induction of autophagy − all of which resulted in increased survival rates of these flies. Taken together, our results provide *in vivo* evidence for a neuroprotective function of the glycogen synthesis pathway and glycogen.

## RESULTS

### Increased levels of glycogen and GS activity in the brain of the fly model for HD

**Fig. 1.**
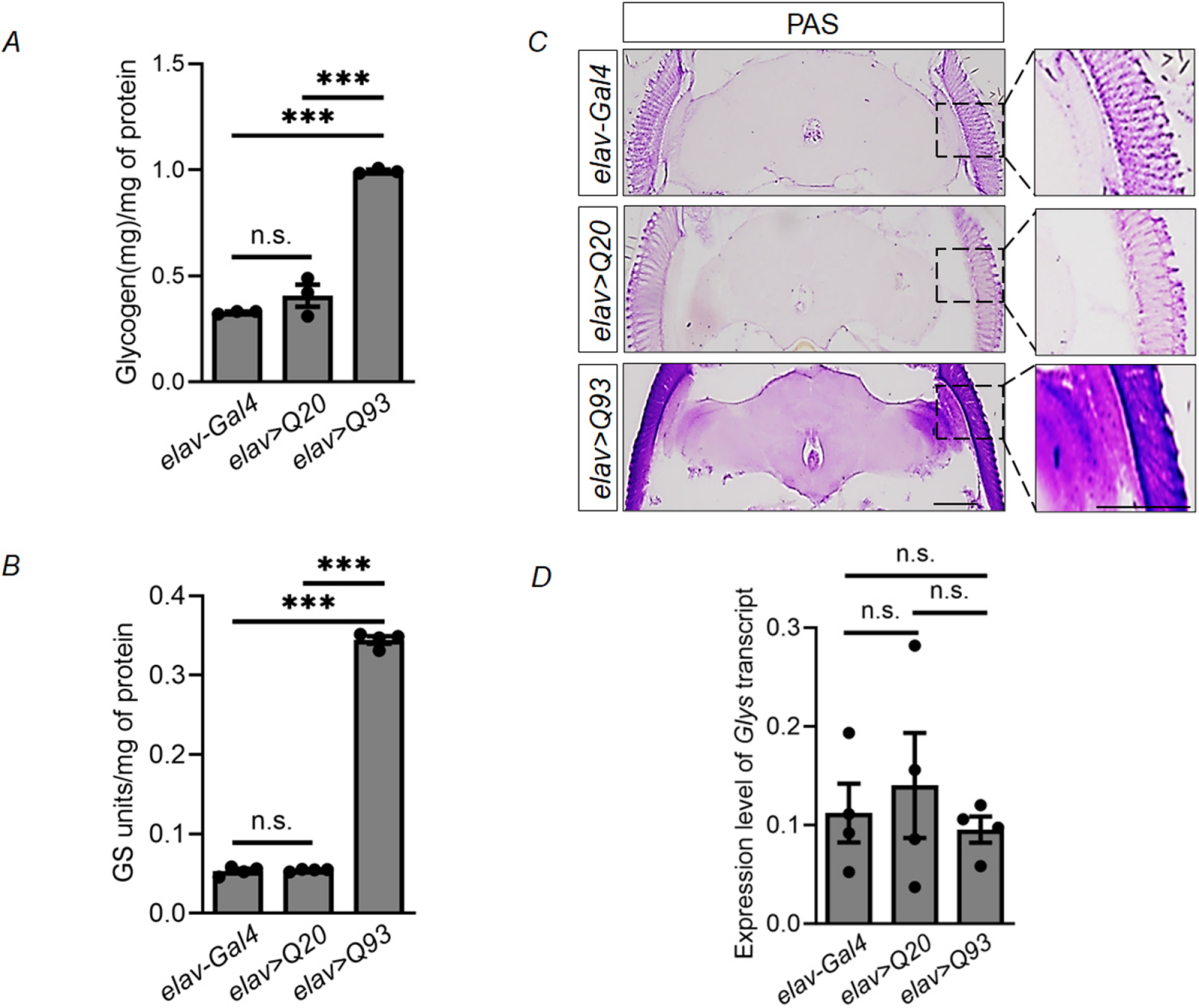
**Increased glycogen levels and GS activity in the brain of HD model flies.** (A,B) Bar diagrams showing absolute glycogen levels (A) and the overall GS activity (B) in head lysates of 1-week-old flies of the indicated genotypes, with *elav-Gal4* and *elav>Q20* serving as control genotypes. Values for all were normalized to the total protein content. (C) Representative bright-field images for PAS staining showing glycogen distribution in head sections of 1-week-old flies of the indicated genotypes. Images on the right are magnified views of the boxed areas on the left. Scale bars: 100 µm. (D) Bar diagram showing the transcript levels of GS (*Glys*) in head-extracts of 1-week-old flies of the indicated genotypes. GS transcript levels were normalized to that of the housekeeping gene *Rpl32*. Each bar represents the mean±s.e.; ***P<0.001, n.s., not significant; *n*=3-4.

We have previously reported increased glycogen levels and GS activity in the cellular and mouse models of HD (Rai et al., 2018). To check if these changes are generic responses towards HD pathology, we used the UAS-Q93 *Drosophila* model of HD as described by Lin et al., (2019). In these flies (hereafter referred to as *elav>Q93*), exon 1 of the human *HTT* gene contains 93 cytosine–adenine–guanine (CAG) repeats that encode the pathogenic polyglutamine (polyQ) expansion, typical for HD. Overexpression of this polyQ expansion is regulated in a brain-specific manner by the pan-neuronal driver *elav-Gal4*. In this current study, *Drosophila* lines *elav-Gal4* and *elav>Q20*, the latter of which expresses the non-pathogenic polyQ expansion of human HTT, served as the controls (see Lin et al., 2019). We estimated the total glycogen content (Fig. 1A) and the activity of GS (Fig. 1B) – i.e. the enzyme responsible for glycogen synthesis−in the fly heads of all three genotypes (*elav-Gal4*, *elav>Q20* and *elav>Q93*). As described for the cellular and mouse models of HD (Rai et al., 2018), there was a significant increase in brain glycogen levels as well as GS activity in the heads of HD flies (*elav>Q93*) compared with those of controls (*elav-Gal4* and *elav>Q20*) (Fig. 1A,B). The increased glycogen levels in the brain tissue were also visualized by using periodic acid–Schiff (PAS) staining, which detects glycogen. We observed the presence of distinct glycogen granules distributing throughout the brain of the HD flies (*elav>Q93*) (Fig. 1C). However, the staining for glycogen was sparse in the case of the controls (*elav-Gal4* and *elav>Q20*) (Fig. 1C). The above results confirmed increased GS activity and concomitant increase in glycogen levels in the brain tissue of the HD fly model. We also checked whether the increased GS activity was due to transcriptional upregulation of the GS gene in the HD fly brain (*elav>Q93*) or not. For this, we measured the transcript levels of *GS* by using quantitative PCR (Fig. 1D). However, no significant difference in *GS* transcript levels was observed across the three genotypes (*elav-Gal4*, *elav>Q20* and *elav>Q93*) studied, suggesting that the increased glycogen level observed in the HD fly is due to post-translational modification or, possibly, allosteric activation of GS (Roach et al., 2012).

### Brain-specific overexpression of GS increases the overall lifespan of HD flies

**Fig. 2.**
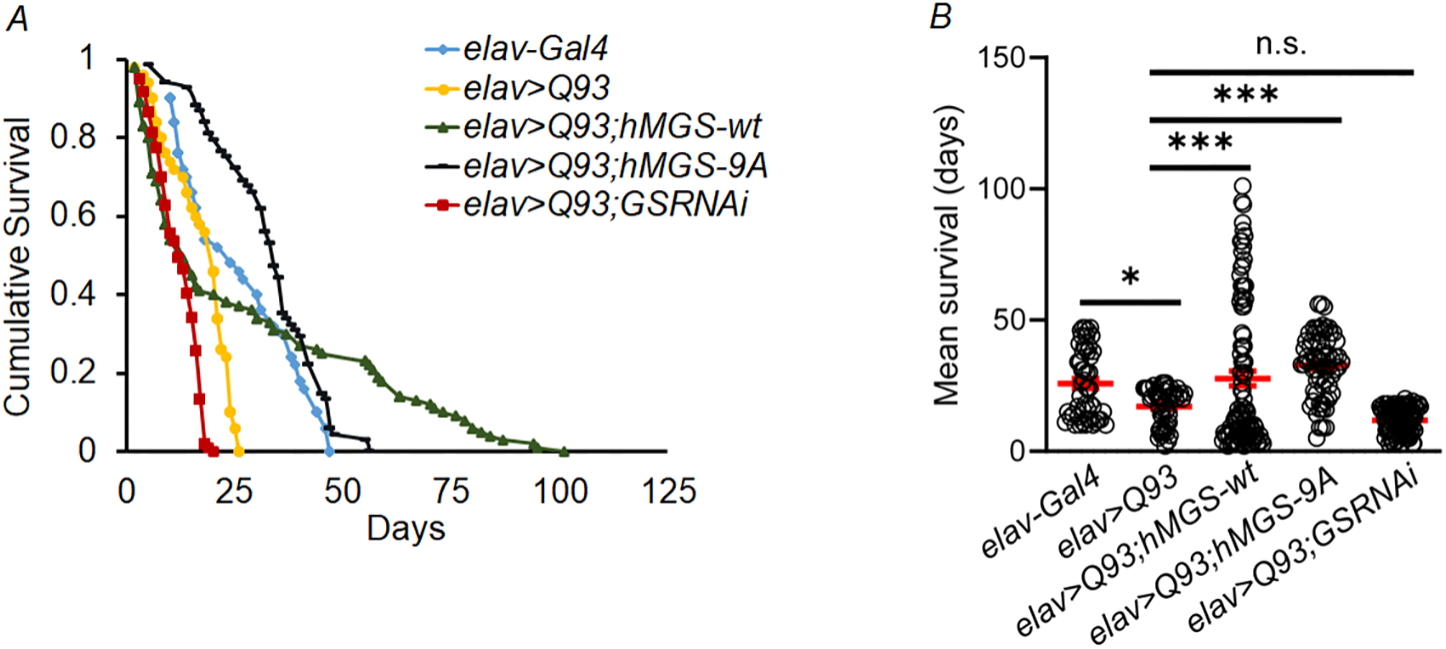
**GS overexpression extends the overall lifespan in HD model flies.** (A) Kaplan–Meier survivorship graphs showing cumulative survival in days for *elav-Gal4* (control) flies, or flies that overexpress a pan-neuronal mutant version of Huntingtin alone (*elav>Q93*) or in combination with the wild-type and the constitutively active version of GS (*elav>Q93;hMGS-wt* and *elav>Q93;hMGS-9A*), or flies that overexpress the pan-neuronal mutant version of Huntingtin and in which GS has been knocked down (*elav>Q93;GSRNAi*). Data were analyzed by using the log-rank test (χ^2^=109.72; *P*=0.001). (B) Scatter plot showing the average lifespan of all experimental genotypes as indicated (*n*=120 per genotype); red horizontal lines indicate the mean value. *P<0.05, ***P<0.001, n.s., not significant.

Exposure to physiological stresses, such as oxidative stress (Gusarov et al., 2017; Rai et al., 2018), ER stress (Wang et al., 2013; Lytridou et al., 2020) and proteotoxic stress (Rai et al., 2018), is known to result in increased glycogen levels in the neurons. In all these aforementioned studies, depletion of glycogen resulted in neurotoxicity, suggesting a protective role for glycogen in the stressed neurons. Indeed, overexpression of GS was shown to rescue neuronal death in the cellular model of HD (Rai et al., 2018). We, therefore, wanted to check whether GS activation and increased glycogen level in the brain could play a neuroprotective role in the HD fly model or not. For this, using appropriate transgenic lines, we overexpressed the mutant HTT protein (Q93) with the wild-type version of human muscle glycogen synthase (GYS1, also referred to as hMGS) *Q93;hMGS-wt* or its constitutively active version *Q93;hMGS-9A* (Duran et al., 2012). We also created the RNAi fly line *Q93;GSRNAi*, to partially silence the *Drosophila* homolog of GS and see its effect on the HD phenotype in fly. To ensure brain-specific expression, all these stocks were driven by using *elav-Gal4*. This driver line line also served as control for all experimental genotypes unless stated otherwise. Expression of hMGS protein was confirmed in the *elav>Q93;hMGS-wt* and *elav>Q93;hMGS-9A* lines by using anti-GS antibody that detects mouse and human GS protein (Fig. S1A). A band of relatively little intensity was observed for the *elav>Q93;hMGS-9A* lines (Fig. S1A) compared with that for *elav>Q93;hMGS-wt*. This difference may be attributed to the lower binding affinity of the antibody towards the mutant protein in contrast to the wild-type variant. Quantification of changes in GS activity and glycogen levels in brain tissue of the transgenic lines *elav>Q93;hMGS-wt* and *elav>Q93;hMGS-9A* was performed using biochemical methods as described in Materials and Methods and as shown in Fig. S1B,C. The efficiency of RNA interference (RNAi) in partially silencing the endogenous GS in the *elav>Q93;GSRNAi* line was confirmed through quantitative PCR (Fig. S1D) and glycogen estimation (Fig. S1E) (see Materials and Methods). Reduced lifespan is an established fact in the fly and mouse models of HD (Mangiarini et al., 1996; Krench and Littleton, 2013). Therefore, we sought to evaluate the effect of GS and its variant on the overall lifespan in the HD lines (*elav>Q93;hMGS-wt*, *elav>Q93;hMGS-9A*, and *elav>Q93;GSRNAi*). As reported previously (Mangiarini et al., 1996; Krench and Littleton, 2013), we also observed shortened lifespan in HD flies (*elav>Q93*) compared to controls (*elav-Gal4* and *elav>Q20*) (Fig. S2, Fig. 2A, B; Table 1). Intriguingly, *elav>Q93;hMGS-wt* initially exhibited a sharp decline in survival (50% survival at day 10) compared to *elav>Q93* (70% survival at day 10), *elav>Q93;hMGS-9A* (94% survival at day 10) and *elav>Q93;GSRNAi* (56% survival at day 10). Nevertheless, the survival rate for *elav>Q93;hMGS-wt* plateaued and lasted longer (12% survival at day 70) after a dip at day 16, which is in contrast to that of *elav>Q93* and *elav>Q93;GSRNAi*, which exhibited a sharp drop (10% survival at day 24 and day 17, respectively). As previously reported for GS transgenic flies by Duran et al. (2012) and Sinadinos et al. (2014), we also initially attributed the steep decline in the survival of *elav>Q93;hMGS-wt* to a possible feedback regulation of wild-type GS. This effect, however, may diminish as HD progresses in these flies, leading to a decline in these inhibitory feedback mechanisms. Indeed, a decrease in the activity of glycogen synthase kinase 3 beta (GSK3B) and other GS-negative regulatory kinases has been observed in HD mouse models (Fernández-Nogales et al., 2015). The dissimilar lifespan trend in HD flies that constitutively express GS (*elav>Q93;hMGS-9A*) and are immune to such feedback regulation, provides additional support for this hypothesis. Although we observed a reduction in the survival of HD flies comprising GS knockdown (*elav>Q93;GSRNAi*), this reduction was not statistically significant when compared with *elav>Q93* (Fig. 2A,B; Table 1). The above results suggest that GS activation is sufficient to rescue the mutant Htt-induced reduction in the survival of HD flies, confirming a neuroprotective role of GS in HD pathology.

**Table 1.**
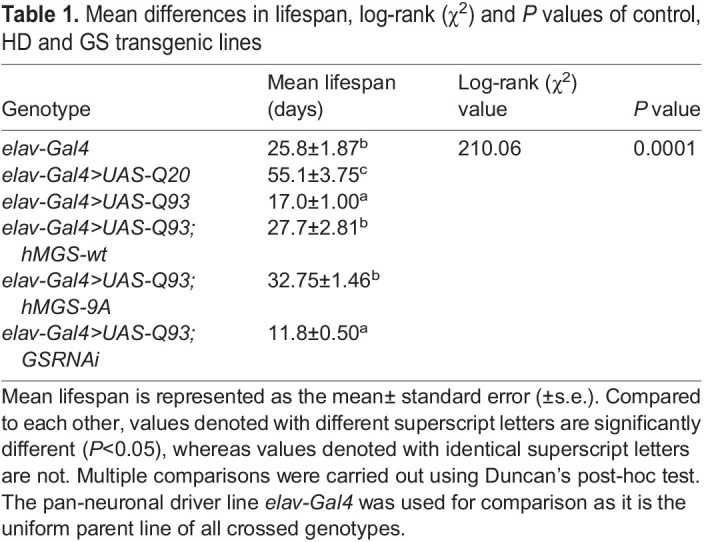
Mean differences in lifespan, log-rank (χ^2^) and *P* values of control, HD and GS transgenic lines

### Brain-specific overexpression of GS rescues photoreceptor degeneration, improves locomotor deficits and augments fitness traits in HD model flies

**Fig. 3.**
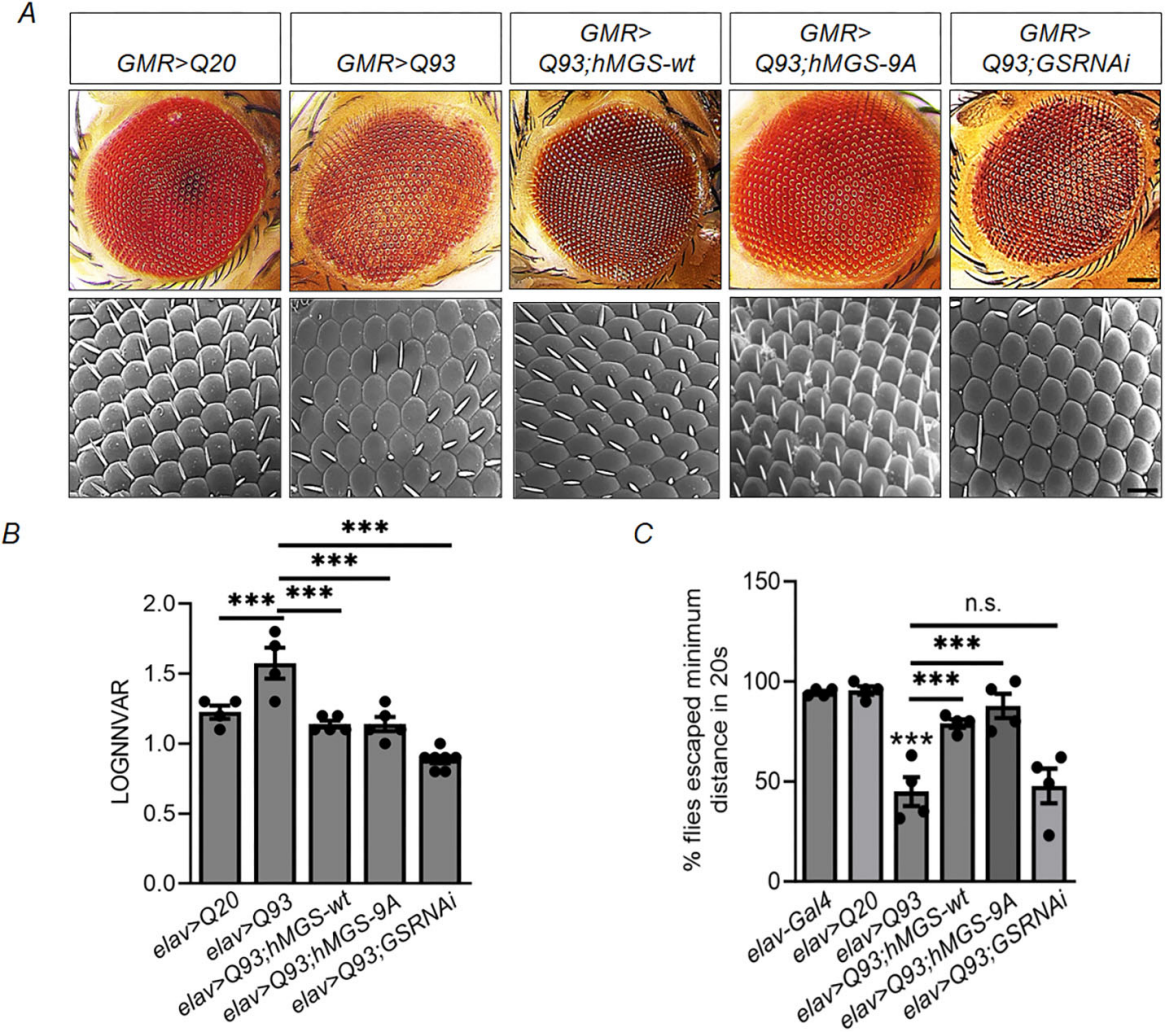
**Enhanced glycogen synthesis alleviates neurodegeneration in HD model flies.** (A) Representative bright-field (top) and scanning electron microscopy (SEM; bottom) images of the eye of 1-week-old flies of the indicated genotypes, with *GMR>Q20* serving as control genotype. Scale bars: 100 µm (bright-field), 50 µm (SEM); *n*=3−4. (B) Quantification of the retinal degeneration in all the genotypes. Each value represents the mean logarithm of nearest neighbor variance (LOGNNVAR)±s.e.; ***P<0.001, n.s., not significant; *n*=3-5 for each group. (C) Quantification of the negative geotaxis behavior (locomotory fitness) of 1-week-old flies of the genotypes indicated. Bar graphs show the number of flies (in %) that escaped the minimum distance within 20 s. *elav-Gal4* served as control for *elav>Q20* and HD fly (*elav>Q93*) lines. HD and GS-compound lines were compared to the HD control line (*elav>Q93*). Each bar represents the mean±s.e.; ***P<0.001, n.s., not significant; *n*=40 for each group.

In addition to the reduced lifespan, HD flies are also known to show neural degeneration, leading to loss of photoreceptors (Kumar et al., 2014) – a rough-eye phenotype (Singh et al., 2017) – and progressive impairment of locomotion (Tamura et al., 2011). Given that GS activation had extended the lifespan of the HD flies, we wanted to check whether increased GS activity and glycogen content in the brain can also ameliorate the above-mentioned neurodegenerative phenotypes in HD flies. For this, we used the negative geotaxis assay as described by Feany and Bender (2000) to measure changes in motor activity in transgenic flies. We found that 1-week-old HD flies (*elav>Q93*) displayed reduced ability to climb compared to control flies (*elav-Gal4* and *elav>Q20*) (Fig. 3C). However, although this deficit in motor abilities could be partially rescued in age-matched HD flies overexpressing GS (*elav>Q93;hMGS-wt* and *elav>Q93;hMGS-9A*) (Fig. 3C), the locomotion defect persisted in HD flies carrying a GS loss-of-function (*elav>Q93;GSRNAi*), with no significant difference compared with flies expressing mutant Htt alone (*elav>Q93*) (Fig. 3C). Next, we examined the external morphology of the eye and the rhabdomere morphology to estimate the progressive loss of photoreceptor and ommatidial degeneration. The external eye of the HD flies (*GMR>Q93*) displayed extensive loss of pigmentation and retinal architecture compared to that of controls that expressed the non-pathogenic Htt variant (*GMR>Q20*) (Fig. 3A). Moreover, the pseudo pupil appeared severely degenerated in 1-week-old HD flies (*GMR>Q93*) when compared to age-matched controls (*GMR>Q20*) (Fig. S3). On the one hand, age-matched HD flies with brain-specific GS expression (*GMR>Q93;hMGS-wt* and *GMR>Q93;hMGS-9A*) showed, to a great extent, rescue of the progressive pigment loss and rough-eye phenotype (Fig. 3A), and improvements in the rhabdomere organization (Fig. S3). On the other hand, compared to the HD flies (*GMR>Q93*), GS knockdown in HD flies (*GMR>Q93; GSRNAi*) did not yield significant differences regarding these features (Fig. 3A, Fig. S3). The eye phenotype of the mutant line *elav>Q93* – characterized by irregularly spaced ommatidia, suggesting degeneration – exhibited significantly elevated values of the mean logarithm of nearest neighbor variance (LOGNNVAR) (Diez-Hermano et al.; 2015) compared to those in control line *elav>Q20* (Fig. 3B). Both *elav>Q93;hMGS-wt* and *elav>hMGS-9A* exhibited LOGNNVAR values comparable to those of control, suggesting consistency in accordance with the depicted images (Fig. 3A). Interestingly, when compared to the control group, experimental group *elav>Q93;GSRNAi* exhibited a statistically significant decrease in LOGNNVAR values. This decrease was accompanied by the presence of irregularly clustered ommatidia, which is indicative of degeneration, as shown in Fig. 3B. Taken together, our results establish the role of GS in ameliorating the neurodegenerative changes in HD flies.

Gonadal atrophy and alterations in the hypothalamic–pituitary–gonadal axis have been reported in HD models, and are thought to underlie the infertility seen in HD mouse models (Van Raamsdonk et al., 2007; Du et al., 2015). Therefore, we wanted to check whether fecundity is affected in HD flies and whether activation of GS can rescue this phenotype. For this, we measured the overall fecundity in HD flies (*elav>Q93*) and in HD flies overexpressing GS (*elav>Q93;hMGS-wt* and *elav>Q93;hMGS-9A*). In *elav>Q93* flies, fecundity was, indeed, reduced compared to that of the control line (*elav-Gal4*) (Fig. S4A). Intriguingly, fecundity significantly improved in both GS-overexpressing lines (*elav>Q93;hMGS-wt* and *elav>Q93;hMGS-9A*) (Fig. S4A). GS knockdown in the HD flies (*elav>Q93;GSRNAi*), however, displayed no further decrease in fecundity when compared to control HD flies (*elav>Q93*). We also monitored the development time, i.e. egg-to-larva-to-pupa-to-adult stage, in all genotypes. With 10.6 days, HD flies (*elav>Q93*) showed a decreased development time compared to 11.8 days for control lines (*elav-Gal4* and *elav>Q20*). Experimental lines *elav>Q93;hMGS-wt* and *elav>Q93;hMGS-9A*, however, showed standard development times (11.8 days and 12 days, respectively) that were comparable to those of control lines (Fig. S4B). In contrast, experimental line *elav>Q93;GSRNAi* showed a significantly longer development time (14 days) for which the longest time (7.3 days) was spent in the egg stage of development. This longer hatching time in *elav>Q93;GSRNAi* indicates a disadvantage to the larval competitive ability that is essential to concomitantly increase the larval feeding rate and to possibly allocate resources during pre-adult stage, which can be utilized to increase the survival rate or other trade-off requisition during adult life (Venkitachalam et al., 2022).

### GS overexpression reduces levels of oxidative stress evident in the brain of HD model flies

**Fig. 4.**
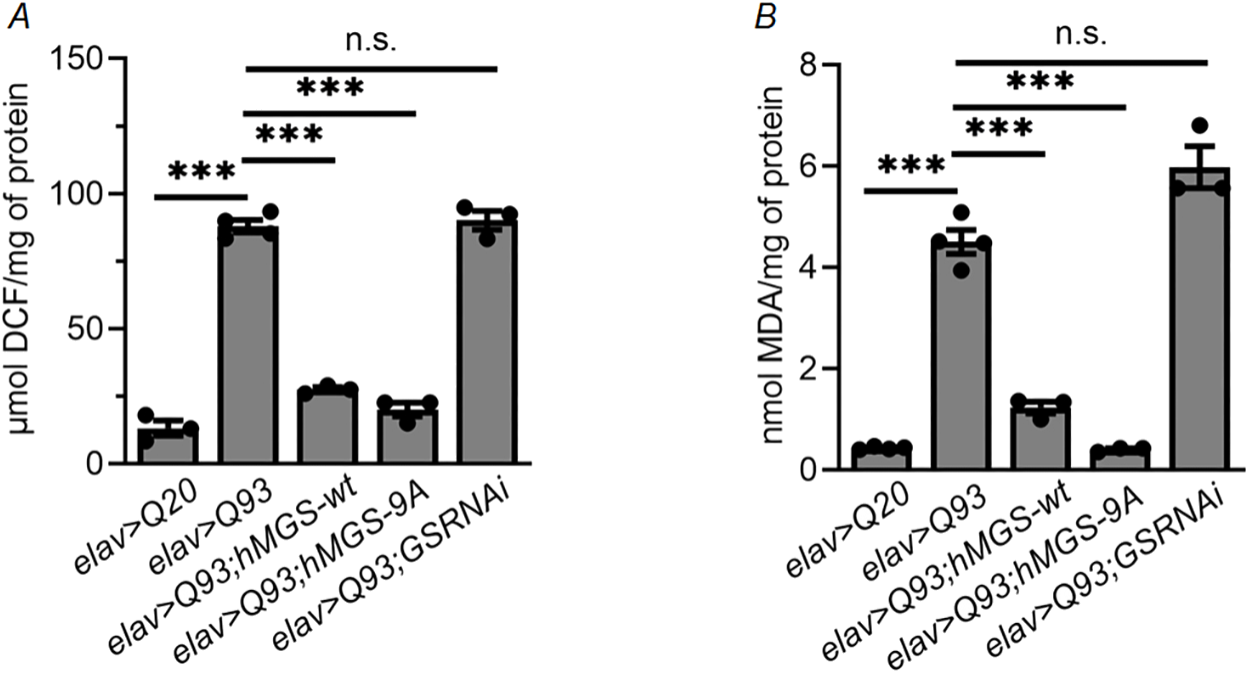
**Elevated glycogen levels reduce oxidative stress in HD model flies.** (A,B) Quantification of reactive oxygen species (ROS) levels by measuring the amounts of dichlorofluorescein (DCF) in μmol (A) and of lipid peroxidation levels by measuring the amounts of malondialdehyde (MDA) in nmol (B) per mg of protein. Analyzed were the heads of 1-week-old flies of the indicated genotypes. For both assays *elav>Q20* served as control. Each bar represents the mean value±s.e.; ***P<0.001, n.s., not significant; *n*=6.

Increased levels of oxidative stress and oxidant-induced damage to cellular biomolecules, possibly due to the cytotoxicity conferred by the mutant Htt protein, have been reported in both the mice and fly models of HD (Browne et al., 1999; Kumar and Ratan, 2016). Given that GS is able to quench increased levels of ROS by enhancing the activity of antioxidant enzymes in the cellular model of HD (Rai et al., 2018), we next investigated whether co-expression of GS and HD can induce a similar effect *in vivo*. To evaluate this, we have employed two different approaches: (i) the DCF-DA method (Black and Brandt, 1974) to quantify relative ROS levels and (ii) the TBARS assay (Ohkawa et al., 1979) to estimate lipid peroxidation levels in fly heads. We observed increased ROS levels and lipid peroxidation status in flies expressing mutant Htt (*elav>Q93*) compared those in the driver line (*elav-Gal4*) (Fig. 4A,B). Higher levels of ROS and lipid peroxidation were also observed in the HD fly line comprising knockdown of GS (*elav>Q93;GSRNAi*), but only to the same extent as those seen in the HD fly line (*elav>Q93*) (Fig. 4A, B). In contrast, HD flies that co-express mutant Htt and either wild-type GS (*elav>Q93;hMGS-wt*) or the constitutively active form of GS (*elav>Q93;hMGS-9A*) displayed relatively low levels of ROS and lipid peroxidation compared to levels in the *elav>Q93* flies (Fig. 4A,B). Thus, we conclude that the GS-mediated reduction in oxidative stress levels in HD flies can potentially rescue the progressive degeneration and extends the lifespan of HD flies.

### GS overexpression reduces the mutant Htt aggregate load in the brain of HD model flies

**Fig. 5.**
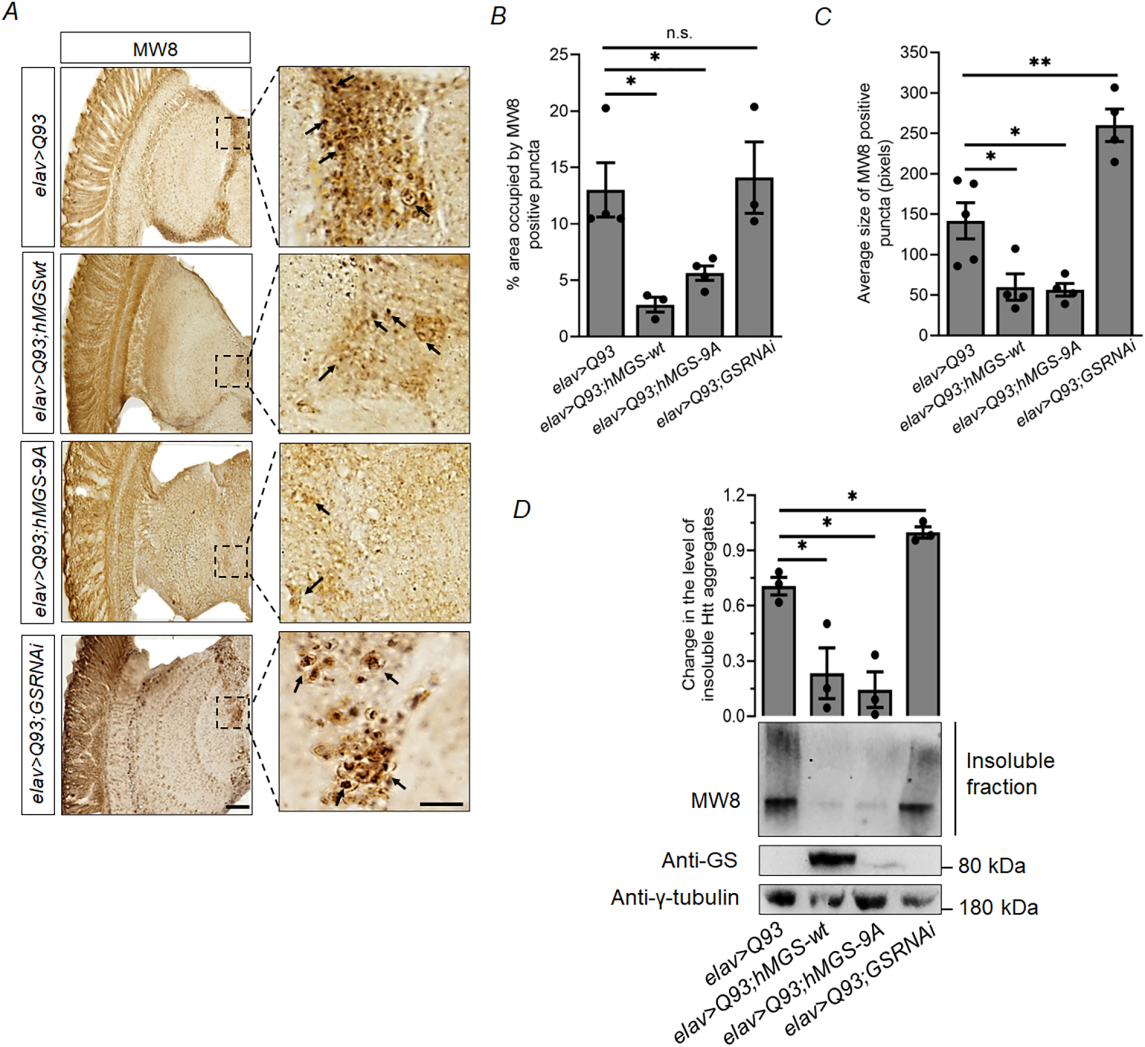
**GS overexpression reduces the aggregate load of mutant Htt in the brain of HD model flies.** (A) Representative bright-field images showing Htt aggregates − detected with antibody MW8 that specifically detects mutant Htt − in the brain of 1-week-old flies of the indicated genotypes. HD flies (*elav>Q93*) were used as control. Images on the right are magnified views of the boxed areas. Arrows indicate Htt aggregate puncta. Scale bars: 50 µm. (B,C) Quantification of Htt aggregates. Bar diagrams showing the area (in %) occupied by MW8-positive puncta (B) and the average size (in pixels) of MW8-positive puncta (C) in the brains of 1-week-old flies of the indicated genotypes. HD flies (*elav>Q93*) were used as control. (D) Analysis of Triton X-100-insoluble high molecular weight Htt aggregates probed against MW8 in whole-head lysates of 1-week-old flies of the indicated genotypes. Top panel: bar diagram showing relative levels of insoluble Htt aggregates obtained by densitometric analysis. Each bar represents the mean value±s.e.; *P<0.05, **P<0.01; *n*=3. Bottom panel: representative immunoblot showing absolute levels of Htt aggregates. γ-tubulin served as the loading control.

The mutant Htt protein contains an abnormal stretch of N-terminal polyglutamine repeats that increases the tendency of the protein to form cytotoxic aggregates (DiFiglia et al., 1997; Reiner et al., 2011). These aggregates act as the causative factor behind pathogenesis and neuronal death, possibly leading to the disease symptoms as described for HD models and human HD patients (DiFiglia et al., 1997; Cha et al., 1999; Reiner et al., 2011). Since overexpression of GS partially rescued the disease phenotype and decreased levels of ROS in the HD fly model, we investigated whether GS can confer neuroprotection by reducing the aggregate load in the fly brain. For this, brain sections of 1-week-old *elav>Q93* HD flies and of the double transgenic lines *elav>Q93;hMGS-wt*, *elav>Q93;hMGS-9A*, and *elav>Q93;GSRNAi* were immunoassayed by using the anti-HTT antibody MW8 that exclusively detects aggregated mutant Htt (Ouk et al., 2016). As shown in Fig. 5A, MW8-positive Htt aggregates were prominent in the brain of HD flies (*elav>Q93*). Nonetheless, a noticeable reduction in the number of MW8-positive aggregate puncta was observed in HD flies with brain-specific overexpression of GS (*elav>Q93;hMGS-wt* and *elav>Q93;hMGS-9A*). In these flies, Htt aggregates appeared to be more diffuse and smaller (*elav>Q93;hMGS-wt* and *elav>Q93;hMGS-9A*) (Fig. 5A-C), suggesting that levels of brain glycogen potentially influence the aggregation properties of mutant Htt protein. In the HD fly model comprising GS knockdown (*elav>Q93;GSRNAi*), size and density of MW8-positive puncta were increased, and puncta were packed more tightly compared with those in flies expressing mutant Htt alone (*elav>Q93*) (Fig. 5A,C). However, no significant difference between the mentioned genotypes was observed in the area occupied by aggregates (Fig. 5B). Therefore, the decision was made to stain the optic lobe by using antibody MW8, as this specific region of the fly brain section consistently exhibited noticeable and significant differences. Furthermore, it is worth noting that retinal degeneration is a prominent characteristic of pathologies associated with HD (see Fig. 3A,B; Fig. S3). In addition, a study conducted by Ragauskas et al. (2014) established a correlation between age-dependent aggregation of Htt protein and retinal degeneration through quantification of the MW8-positive area in the optic lobe of the brain. However, our staining procedure was conducted on entire brain sections of flies, and we did observe the presence of aggregates in all regions (data not shown).

To confirm the aggregation properties of mutant Htt in the fly lines, we used soluble–insoluble fractionation to estimate detergent solubility of aggregates from our experimental genotypes. In this approach – as described by Ochaba et al. (2016) – the high-molecular-weight Htt aggregates trapped in the stacking gel represented the detergent-insoluble aggregates. As shown in Fig. 5B, we observed a marked decrease in the levels of detergent-insoluble Htt aggregates within tissue lysates of HD flies that co-expressed either the wild-type or the constitutively active form of GS (*elav>Q93;hMGS-wt* or *elav>Q93;hMGS-9A*, respectively) as compared to the HD fly (*elav>Q93*) when probed for Htt with MW8. We also observed a significant increase in insoluble high-molecular-weight aggregates in HD flies comprising a GS-knockdown background (*elav>Q93;GSRNAi*) compared to levels in HD flies (*elav>Q93*) (Fig. 5B), suggesting that glycogen build-up either prevents aggregation of mutant Htt or promotes its clearance.

### Overexpression of GS in HD model flies rescues HD pathology by increasing the autophagy flux

**Fig. 6.**
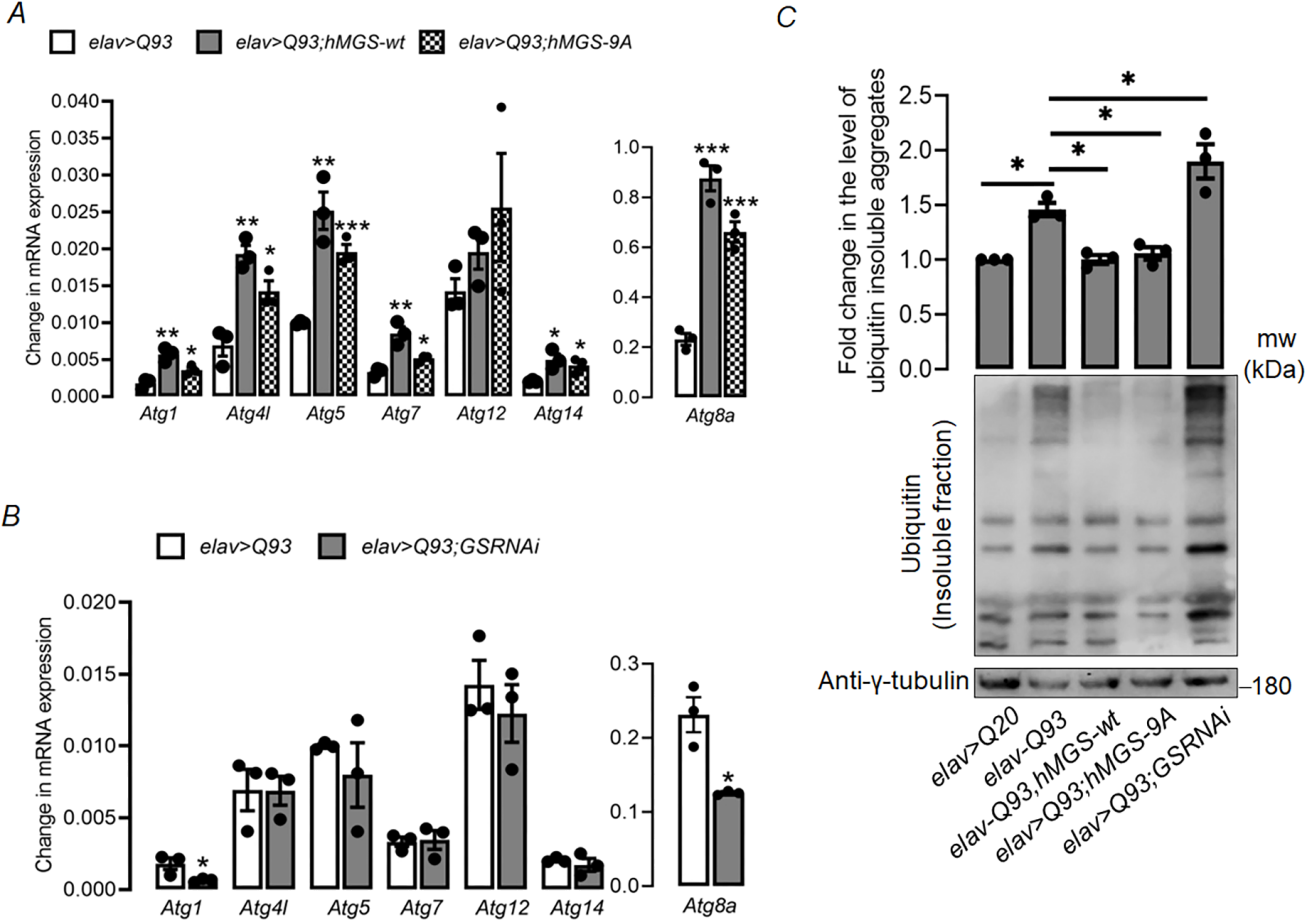
**Increase in glycogen levels enhances the autophagy flux in the brain of HD model flies.** (A,B) Quantification of transcript levels (mRNA expression) of essential autophagy-related genes (Atg1, Atg4l, Atg5, Atg7,Atgg12, Atg14) in whole-head lysates of 1-week-old flies of different genotypes as indicated. (B). Each bar represents the mean±s.e.; *P<0.05, **P<0.01, ***P<0.001; *n*=3. Expression values for all genes were normalized to that of the housekeeping gene *Rpl32*. (C) Analysis of ubiquitin-insoluble aggregates in whole-head lysates of 1-week-old flies of the indicated genotypes. Top panel: bar graph showing the relative fold-change of aggregate levels obtained by densitometric analysis. *elav>Q20* served as the control for *elav>Q93*, whereas all the other genotypes were compared to *elav>Q93,* as it is the uniform parent line of all crossed genotypes. γ-tubulin served as loading control. Each bar represents the mean value±s.e.; *P<0.05; *n*=3. Bottom panel: representative immunoblot showing relative levels of Triton X-100-insoluble ubiquitin aggregates γ-tubulin served as the loading control.

Given our observations that glycogen accumulation in the brain alleviated HD pathology, we next explored the possible mechanism behind this protective effect. Autophagy has been widely implicated as a therapeutic pathway both in *in vitro* and *in vivo* models of HD (Harding and Tong, 2018; Pierzynowska et al., 2018). We have recently shown that GS can induce autophagy and that the GS-mediated reduction of Htt aggregates is autophagy dependent in cellular models of HD (Rai et al., 2018). Therefore, we explored whether brain glycogen accumulation can mitigate HD pathology by increasing also the autophagy flux in the fly model of HD. For this, we measured the expression levels of some essential autophagy-related genes (ATGs) in brains of HD (*elav>Q93*) and control flies (*elav>Q20*), observing significantly reduced expression levels of seven ATGs in HD fly versus control fly brains (Fig. S5). Next, we checked whether GS overexpression in HD flies can restore these expression levels. We observed a significant increase in the transcript levels of most ATGs in HD flies expressing either the wild-type or the constitutively active form of GS (*elav>Q93;hMGS-wt* and *elav>Q93;hMGS-9A*, respectively) (Fig. 6A). In HD flies comprising partially silenced GS (*elav>Q93;GSRNAi*), expression levels of only two ATGs (*Atg1* and *Atg8a*) were significantly reduced as compared to HD control flies (*elav>Q93*) (Fig. 6B).

An increase in the basal autophagic flux is known to remove aggregated and ubiquitylated proteins in cells (Simonsen et al., 2008). Ubiquitin-positive Htt aggregates (Garyali et al., 2009) and a compromised ubiquitin-proteasomal pathways are well-established for HD, and contribute to the disease phenotype (Juenemann et al., 2018). Given the transcriptional activation of ATGs upon GS overexpression in HD flies, we next evaluated the functional significance of the increased autophagy flux by measuring the levels of ubiquitylated proteins in an immunoblot as a readout. As reported previously (Juenemann et al., 2018), HD flies (*elav>Q93*) exhibited increased insoluble poly-ubiquitylated aggregates as compared to control flies (*elav>Q20*) (Fig. 6C). This increase in insoluble ubiquitylated proteins was further enhanced in HD flies upon GS knockdown (*elav>Q93;GSRNAi*) (Fig. 6C). The increased autophagy marker levels in the glycogen-enriched HD fly lines (*elav>Q93;hMGS-wt* and *elav>Q93;hMGS-9A*) (Fig. 6A) corroborated a reduction in the levels of insoluble poly-ubiquitylated proteins as compared to the HD control flies (*elav>Q93*) (Fig. 6C). Thus, the above results indicate that brain glycogen can modify the autophagy process in the phenotype of HD flies.

### Increased brain glycogen levels mitigate lysosomal defects evident in HD model flies

**Fig. 7.**
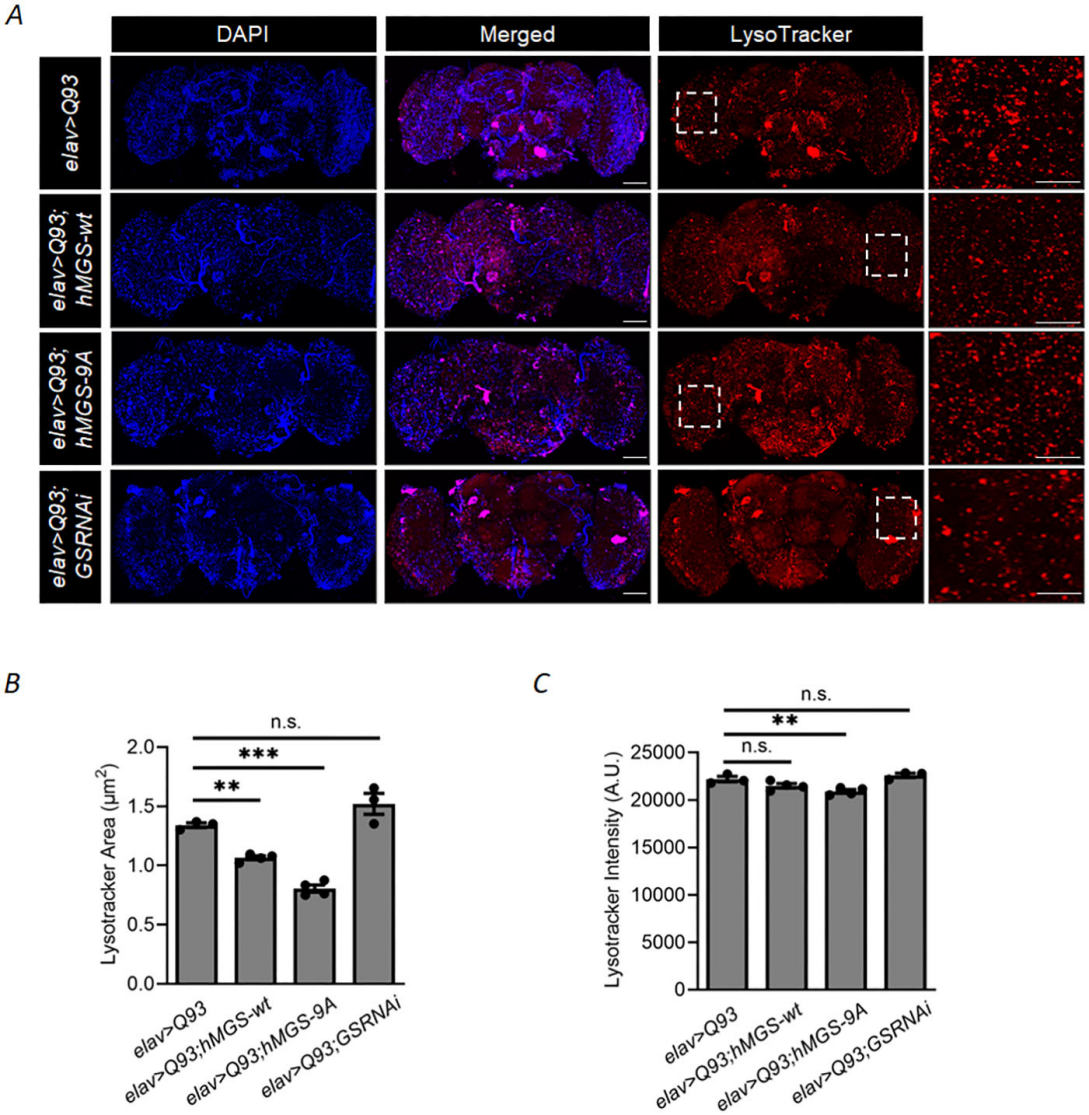
**Increase in brain glycogen levels rescues lysosomal abnormalities in HD model flies.** (A) Representative fluorescence images of brains obtained from 1-week-old flies of the genotypes indicated, stained for lysosomes (red) by using LysoTracker RED. The pan-neuronal driver line *elav-Gal4* was used for comparison as it is the uniform parent line of all crossed genotypes. Nuclei were stained with DAPI (blue). Images on the right are magnified views of the boxed areas within the penultimate column. Scale bars: 60 µm. (B,C) Bar diagrams representing area of lysosome puncta in µm^2^ (B) and signal intensity of lysosome puncta (C) in fly brains of the indicated genotypes. Experimental genotypes were compared to HD control (*elav>Q93*). Each bar represents the mean value±s.e.; **P<0.01, ***P<0.001; n.s., not significant, *n*=3-4.

Apart from the defects in the autophagy process, compromised lysosomal functions are also thought to underlie neurodegeneration in HD (Malik et al., 2019). For instance, increased lipid accumulation in the lysosomes has been reported in HD patients (Tellez-Nagel et al., 1974), which renders the lysosomes incapable of degrading substrates, thus, resulting in the accumulation of toxic proteins. Therefore, we wanted to analyze lysosomal function in HD flies and the effect of GS on this process. For this, we stained the brains of all fly genotypes by using LysoTracker RED, and visualized the lysosomal structure and signal intensity. Interestingly, we observed abnormally enlarged lysosomes and increased lysosomal area in the brain of the HD fly (*elav>Q93*), which was not observed in the age-matched brain tissues of control fly (*elav>Q20*) (Fig. S6A,B). There was, however, no difference in the LysoTracker signal intensities between the two genotypes (Fig. S6C). A similar structural deformity of the lysosome was also evident in HD flies comprising knockdown of GS (*elav>Q93;GSRNAi*); however, we did not observe a significant difference in the lysosomal area between these two genotypes (*elav>Q93* and *elav>Q93;GSRNAi*) (Fig. 7A,B). In contrast, lysosomal size and area were significantly reduced in HD flies upon GS overexpression (*elav>Q93;hMGS-wt* and *elav>Q93;hMGS-9A*) (Fig. 7A,B), suggesting that activation of GS and increased glycogen levels in HD brains can restore lysosomal structural deficits in HD flies. We also assessed the signal intensity of LysoTracker RED staining (Fig. 7C). We did not observe a significant difference in the intensity parameter across genotypes, except between the HD fly (*elav>Q93*) and the HD fly expressing the constitutively active GS (*elav>Q93;hMGS-9A*) (Fig. 7C). Here, LysoTracker intensity was decreased in the latter, suggesting increased turnover of lysosomes and, thus, increased induction of autophagy (Fig. 7C).

## DISCUSSION

We have earlier shown that overexpression of GS reduces the cytotoxicity mediated by mutant Htt in a cellular model of HD (Rai et al., 2018). This current study was planned to check whether or not activation of GS and a concomitant increase in glycogen levels within the brain can arrest or ameliorate neurodegeneration in a fly model of HD, thereby rescuing the disease phenotype. Using transgenic fly lines, we demonstrated that overexpression of GS, indeed, ameliorates neurodegeneration and rescues HD phenotype. Further, we noted that the positive effect of GS in the HD model is not restricted to brain functions but also augments life fitness traits, such as fecundity. Finally, we demonstrated that, at the cellular level, activation of GS protects the brain from the mutant protein by suppressing the oxidative stress and increasing the autophagy flux, suggesting a direct role for cellular glycogen in neuroprotection.

Autophagy is a crucial cellular process that is required for energy balance, and defects herein are associated with several neurodegenerative disorders, including HD (Menzies et al., 2006; Nixon, 2013; Moujalled et al., 2021). Indeed, induction of autophagy ameliorates the HD phenotype in model systems (Walter et al., 2016; Martinez-Vicente et al., 2010). Therefore, the neuroprotection exerted by overexpressed GS in our fly model of HD is likely to be through the induction of autophagy. Consistent with this view, we found GS-induced upregulation of autophagy-related genes and effective clearance of cytotoxic mutant Htt protein in fly lines overexpressing GS. While we have not dissected the mechanism through which GS might activate the autophagy process, it is intriguing to note that a direct interaction between GS and the autophagosome membrane protein Atg8a has been demonstrated (Zirin et al., 2013), suggesting a – possible direct – role for GS in the autophagy process. Alternatively, the GS-mediated increase in glycogen might indirectly induce autophagy. As for glycogen, a positive effect of saccharides, such as trehalose, in inducing autophagy in the HD mouse model has been reported (Tanaka et al., 2004). It is equally possible that, in HD flies, increased levels of glycogen prevent or delete the aggregation of the mutant Htt by exerting a macromolecular crowding effect (Mittal and Singh, 2014; Rai et al., 2018), thereby helping the autophagy process for their effective clearance. In this regard, it is worth noting that GS overexpression rescued the morphology of large lysosomes, as seen in the HD flies. Large lysosomes are associated with abnormal lipid metabolism and accumulation of glucosylceramide within them (Kinghorn et al., 2016). We, therefore, speculate that shifting the cellular energy hub towards glycogen has reduced the lipid accumulation within lysosomes. Alternatively, GS and glycogen metabolic pathways might have modulated the factors regulating the metabolism of glycosylceramide. Nonetheless, our current study offered insight into the role of glycogen metabolic pathways in auto-lysosome functions and their role in neuronal survival under physiological stress.

Cytotoxic mutant Htt, in addition to blocking the proteolytic processes, is known to induce oxidative stress (Túnez et al., 2011; Zheng et al., 2018). Our observations that increased brain glycogen ameliorates the HD phenotype in flies indicate that brain glycogen could bring down levels of ROS within the cell. Indeed, an oxidant scavenging role of glycogen contributing to increased survival benefits has been reported in *Caenorhabditis elegans* (Gusarov et al., 2017). Reduction in the level of oxidative stress, a factor known to increase aggregate interaction and assembly (Mitomi et al., 2012), could also help to reduce mutant Htt levels. We suggest that GS and glycogen function at multiple levels in reducing the load of mutant Htt protein, and further work is required on individual pathways.

One of the intriguing observations of our current study is the effect of overexpressed GS on the fitness traits in HD flies, specifically regarding the fecundity rate. HD animal models are known to exhibit deterioration in fertility parameters (Mangiarini et al., 1996). However, the exact mechanism by which an increase in brain glycogen levels causes changes in the rate of fecundity in HD animal models is unknown. GS-induced functional restoration of the neuroendocrine–gonadal axis in HD flies could be one explanation. Indeed, defects in neuroendocrine functions have been identified for HD (Saleh et al., 2009; Bartlett et al., 2016) and should be investigated further. Another possibility is glycogen-mediated longevity enhancement in HD flies. Shorter development time has been associated with lower pre-adult survival (Prasad et al., 2000), which is true in the case of HD flies (*elav>Q93*) in our study. Furthermore, longer development time allows for better resource acquisition to extend survival or maintenance of other fitness traits, as observed in our experimental lines. Indeed, it has been shown that longer development time is positively related to longevity (Buck et al., 2000). It should be noted, however, that a difference in the fertility rate has only been shown in HD model organisms, not in humans diagnosed with HD. One possible explanation is that the HD phenotype, with varying CAG repeats, follows anticipation in humans. In contrast, the CAG repeats in HD models are more or less stable, with no significant differences regarding the onset age of the disease. Nonetheless, our current findings offer insight into the observed defects in HD model organisms.

Several studies have reported the presence of glycogen in the autopsied brain of patients with neurodegenerative disorders, such as Alzheimer' and Parkinson' disease (Mann et al., 1987; Trivedi et al., 2003; Rohn, 2015; Rai et al., 2018), and in brains of unaffected aged individuals (Augé et al., 2017; Riba et al., 2021a,b). Our observations are strong enough to suggest that physiological stress is one of the triggers for GS activation, and the glycogen accumulation seen in these conditions could represent one of the readouts of the neuronal stress response mechanism. Indeed, increased levels of glycogen in neuronal cultures exposed to physiological stressors have been reported in the literature (Wang et al., 2013; Saez et al., 2014; Gusarov et al., 2017; Rai et al., 2018; Lytridou et al., 2020; Pairor et al., 2021). Therefore, overexpression of GS in the HD fly line would have supplemented the endogenous GS-initiated autophagy process, better protecting neurons from cytotoxicity and extending their lifespan. Moreover, given the similar amount of GS transcript levels between control and HD flies – despite high glycogen levels in the latter – and given the mild phenotypes we observed in HD flies comprising knocked down GS, modulation of endogenous GS protein by using more-advanced expression systems, such as the auxin-inducible GAL4-compatible system described by McClure et al. (2022), could be a way to further uncover the role of GS in HD pathology. Nonetheless, the glycogen synthesis pathway can, potentially, be considered a therapeutic target for the treatment of HD and other neurodegenerative disorders.

## MATERIALS AND METHODS

### Reagents and antibodies

The following chemicals were purchased from Sigma Aldrich: amyloglucosidase (catalog no. A7420), glucose-6-phosphate (catalog no. 10127647001), pyruvate kinase/lactate dehydrogenase enzyme solution (PK/LDH, catalog no. P0294), 2′,7′-dichlorofluorescein diacetate (DCF-DA; catalog no. D6883), 2-thiobarbituric acid (catalog no. T5500), Triton X-100 (catalog no. T8787). Other reagents used are: uridine 5′-diphosphoglucose disodium salt hydrate (UDPG.2Na hydrate, catalog no. 20964, Sisco Research Laboratories Pvt. Ltd., India), periodic acid (catalog no. 19184, Thermo Fisher Scientific India Pvt. Ltd.), Schiff' reagent (catalog no. 141407; Thomas Baker Chemicals Pvt. Ltd. India), TRIzol (catalog no. 15596026, Thermo Fisher Scientific), RiboLock RNase inhibitor (catalog no. EO0381, Thermo Fisher Scientific), reverse transcriptase (catalog no. EP0352, Thermo Fisher Scientific), Oligo (dT)_18_ primer (catalog no. SO132, Thermo Fisher Scientific), 1× protease inhibitor cocktail (catalog no. P2714, Sigma-Aldrich), 1× phosphatase inhibitor cocktail (catalog no. 4906845001; Roche Products India Pvt. Ltd.) and nitrocellulose membrane (catalog no. 1620112; Bio-Rad Laboratories India Pvt. Ltd.).

The following antibodies were used in the study: mouse monoclonal anti-γ-tubulin (1:5000; catalog no. T6557, Sigma Aldrich), anti-glycogen synthase (#3893; IB; 1:1000; Cell Signaling Technology), mouse monoclonal anti-HTT (1:700; MW8, Developmental Studies Hybridoma Bank), mouse monoclonal anti-ubiquitin (clone FK1; 1:1000; catalog no. BML-PW8805, Enzo Lifesciences). For cell staining, the red-fluorescent dye LysoTracker Red DND-99 (catalog no. L7528; Invitrogen, Thermo Fisher Scientific) was used.

### Transgenic fly stocks and culture

The fly lines expressing the 20 polyglutamine (polyQ) repeats (*UAS-Q20*) (Kazemi-Esfarjani and Benzer, 2000), the mutant line expressing the 93 polyQ repeats encoded by exon 1 of the *HTT* gene (*UAS-httex1Q93 or UAS-Q93*) (Kazemi-Esfarjani and Benzer, 2000), the pan-neuronal driver line *elav-Gal4^C155^* (Yao et al., 1993) and the eye-specific driver line that expresses in the eye-imaginal discs of the larvae and in the adult eye (*GMR-Gal4*) (Kumar et al., 2014) were kindly provided by Prof. Pradip Sinha (IIT Kanpur, India). Transgenic lines expressing the wild-type form of GS (*UAS-hMGS-wt*), the constitutively active form (*UAS-hMGS-9A*) and the GS-knockdown line (*UAS-GSRNAi*) were a kind gift from Prof. Joan J. Guinovart (IRB, Barcelona, Spain), and have been described in previous studies (Duran et al., 2012; Sinadinos et al., 2014; Sheshadri et al., 2021). Males of all the transgenic lines used in the study were crossed with virgins of *elav-Gal4^C155^* and *GMR-Gal4* to drive their expression in the fly brain and the eye-imaginal discs, respectively.

Mutant Htt protein (Q93) was expressed with the human muscle glycogen synthase (GLYS1, also referred to as hMGS) by using standard recombination and segregation methods to create three additional stocks. These three stocks are (i) Q93 with wild-type isoform of hMGS (*+/+; Q93/CyO;hMGS-wt/MKRS*); (ii) Q93 with the constitutively active version of hMGS (*+/+; Q93/CyO;hMGS-9A/MKRS*); (iii) Q93 with the RNAi of GS (*+/+; Q93/CyO; GSRNAi/GSRNAi*). The *elav-Gal4^C155^*, *elav>Q20* and *GMR>Q20* lines served as controls as indicated. All flies were maintained on standard cornmeal–agar medium at 25±1°C under constant light-dark cycle and 70%-80% humidity. Males of all experimental genotypes were crossed with the virgins of *elav-Gal4^C155^* to drive their expression pan-neuronally, and equal numbers of male and female F1 progeny were used for all the analysis and assays. Transactivation was carried out at 25±1°C.

### Glycogen synthase activity

Glycogen synthase (GS) activity was measured using Danforth's spectrophotometric method as previously described (Danforth, 1965; Sheshadri et al., 2021). Briefly, fly whole-head lysates were added to a reaction mixture comprising 48 mM Tris pH 8.2, 12.4 mM MgCl_2_, 1 mM EDTA, 2.4 mM 2-mercaptoethanol, 3.63 mM UDPG.2Na hydrate and 9.7 mM glucose-6-phosphate. A decrease in the absorbance at 340 nm with the addition of the enzyme was measured for 3 min.

### Glycogen estimation

The glycogen content in the fly heads was measured using the protocol described earlier (Sheshadri et al., 2021). Briefly, for each genotype twenty fly heads were homogenized in 100 µl of 30% KOH solution and boiled at 100°C for 20 min. Eighty µl of this sample was spotted on a 2×2 cm Whatman filter paper (#31-ET CHR). The spotted sample was given three consecutive washes with ethanol (66%) and then dried overnight. The dried filter paper was further treated with the enzyme amyloglucosidase (0.5 mg/ml in 0.2 M sodium acetate buffer pH 4.8) to release glucose. This free glucose was measured using the glucose colorimetric assay kit (catalog. no.: 120200, ERBA Diagnostics Mannheim GmbH, Mannheim, Germany) and normalized to the total protein content.

### Tissue embedding and histology

Fly heads were embedded in paraffin as previously described (Drobysheva et al., 2008). Briefly, 20 fly heads for each genotype were fixed in freshly prepared Carnoy' fixative (ethanol:chloroform:glacial acetic acid at the ratio 6:3:1) overnight at 4°C. The heads were then dehydrated using graded ethanol series (100%, 95%, 70%, 50%, 25%) and embedded in paraffin. Embedded fly heads were sectioned at 5 µm and used for glycogen staining and histochemical assays.

### Periodic acid–Schiff (PAS) staining

Periodic acid–Schiff (PAS) staining was performed on the fly heads as performed earlier (Sinha et al., 2021). For PAS staining, paraffin-embedded fly heads of all genotypes were deparaffinized and hydrated using a graded ethanol series (100%, 95%, 80%, 70%, 50%, and 25%). The sections were then oxidized using periodic acid (1%w/v) for 20 min at room temperature, followed by washes with autoclaved water. After oxidation, sections were treated with Schiff reagent for 20 min at room temperature and then rinsed in tap water. After the staining, sections were dehydrated, mounted in DPX and observed under the Nikon ECLIPSE C*i-*L brightfield microscope at 10× magnification.

### RNA extraction and quantitative real-time PCR

RNA extraction and real-time PCR to analyze gene expression was performed as previously described (Sheshadri et al., 2021). Briefly, total RNA was isolated from 20–25 fly heads for each genotype by using Trizol reagent according to the manufacturer' protocol (Life Technologies, India). cDNA was synthesized from 1 µg of total RNA using random primers; the efficacy was verified by amplifying the housekeeping gene *Rpl32*. Real-time PCR was performed using the Luna Universal qPCR master mix (New England BioLabs) and a CFX-96 real-time PCR machine (Bio-Rad Laboratories, India). The primer sequences used in the study are listed in Table S1.

### Lifespan analysis

Freshly eclosed flies were collected and transferred (on the day of eclosion) to glass vials containing 10 ml of standard cornmeal–agar fly medium. Flies were transferred to fresh food vials every alternate day for 60 days and, after that for every 3-5 days, depending on medium condition. Flies dying during survivability assessment were not replaced. Survival of flies was monitored daily until all had died (Sheshadri et al., 2021). For each genotype studied, six replicate experiments were set up with 20 flies in each vial. The mean lifespan of flies was calculated for all the groups.

### Negative geotaxis

Negative geotaxis assay of flies was employed to assess their locomotory ability. Ten flies for each genotype were used in four to six replicate experiments. Flies were transferred to a vertical column (25 cm long and 1.5 cm in diameter). Flies were gently tapped thrice to the bottom of the column; flies that reached the top of the column and those that remained at the bottom were counted separately. Results were expressed as the number of flies that escaped beyond the minimum distance in the 20 s interval (Feany and Bender, 2000; Sheshadri et al., 2021).

### Ommatidia imaging, pseudopupil analysis and scanning electron microscopy

Adult fly heads of all genotypes (7–10 days old) were fixed on glass slides using nail polish. The compound eye phenotype for at least 5–7 flies for each genotype was examined under a stereomicroscope (Leica-M205FA), and one representative acquisition was presented for reference (Singh et al., 2017). Pseudopupil analysis for all the experimental lines was carried out as described previously (Kumar et al., 2014). Briefly, for each genotype, flies aged 7–10 days were decapitated and the rhabdomere arrangement was visualized using the 40× oil objective of the Nikon ECLISPSE C*i-*L brightfield microscope. For scanning electron microscopy (SEM), adult fly heads were processed using the hexamethyldisilazane (HMDS) dehydration method as described previously (Singh et al., 2017). Twenty flies for each genotype were anesthetized with ether and the abdomen was punctured with sharp needles to allow subsequent solvent penetration into the tissue. Samples were kept in a fixative (1% glutaraldehyde, 1% formaldehyde, 1 M sodium cacodylate pH 7.2) and placed on a rocking platform overnight at room temperature. Flies were then dehydrated by using 25%, 50%, 75% and 100% ethanol during 12 h of incubation under gentle rocking. The samples were incubated overnight in ethanol:HMDS (1:1) in the dark. Then, samples were incubated in HMDS alone for 24 h in the dark and vacuum dried. Gold–palladium coating was applied for 60 s at 6 Pa and 40 mA using the JEC-3000FC Auto Fine Coater (JEOL). SEM imaging was carried out using a FEI Quanta 200 scanning electron microscope at 1.5 kV; images were captured at 2500× magnification.

Retinal images of flies were obtained using bright-field microscopy. Each genotype was represented by five images. Quantification of these images was performed using the FLEYE plugin, a freely available tool for image analysis in ImageJ software. The methodology used for image quantification was as previously described by Diez-Hermano et al. (2015). The plugin can identify the user-specified region of interest (ROI) for every image belonging to five distinct genotypes (groups). The plugin subsequently performs image normalization, identifies the maximum pixel values, partitions the region of interest (ROI) into square grid cells, and retrieves statistical and spatial information from each grid cell containing the maxima. The plugin calculates the distance between each individual maximum and its nearest neighbor by using the coordinates of the local maxima. The LOGNNVAR (logarithm of nearest neighbor variance) method was employed to quantify the variations observed in eyes with different levels of degeneration (see Diez-Hermano et al.; 2015). The LOGNNVAR variable functions across the entirety of the region of interest (ROI). In a wild-type eye (control; *elav>Q20*), it is anticipated that the nearest neighbor distances will exhibit greater similarity to one another compared to a degenerated eye (mutants). The degenerated eye, having lost its regularity, is characterized by higher variance in the distances observed.

### Fecundity and development time

The average daily fecundity and the durations of egg-to-adult developmental stages (egg, larva, pupa) for all the experimental groups were carried out as described previously (Sheshadri et al., 2021). Briefly, for measuring fecundity, freshly eclosed virgin flies were collected, sexed and transferred to separate glass vials containing 10 ml cornmeal–agar medium on the same day of eclosion. To determine fecundity, ten unmated males and ten virgin females were added to a medium-containing vial. Every day, the pair was transferred to a new medium-containing vial, and the number of eggs laid on the previous day was quantified using a stereomicroscope. Fecundity assays were conducted over a period of 12 days; dead males were substituted throughout the duration of the assay. For this, a distinct cohort of male flies, sourced from an identical batch of cultures, was concurrently preserved. For each line, three vials, each containing ten pairs of flies, were prepared. The average daily fecundity, which represents the total number of eggs laid by one female per day during a specific time period, was determined based on the collected data.

To assess the duration of all stages of development (egg, larva, pupa), a total of 20 eggs of identical age were introduced per designated culture vial. Vials was kept in the vivarium at 22±1°C, a consistent 12:12 light–dark cycle and a humidity level of 70–80%. The duration of each developmental stage was recorded through constant monitoring of vials until there was no eclosion of flies for two consecutive days. Three vials for each genotype were prepared. The duration (in days) of the egg-hatching process, the pupation process for larvae and the eclosion process for pupae were documented for all genotypes.

### Reactive oxygen species measurement

For all the experimental groups reactive oxygen species (ROS) in fly heads were measured using the 2′,7′-dichlorofluorescein diacetate (DCF-DA) method (Black and Brandt, 1974). Briefly, the reaction mixture consisted of 0.1 M Tris-HCl buffer pH 7.4, 100 µl fly head homogenate and 10 µM DCF-DA. The reaction cocktail was incubated for 30 min at room temperature following the fluorometric estimation of 2′,7′-dichlorofluorescein (DCF) with excitation and emission wavelength of 488 nm and 525 nm, respectively. The values were plotted against the standard DCF graph and expressed as µmol DCF formed/min/mg protein.

### Lipid peroxidation estimation

The lipid peroxidation levels in the fly heads of the transgenic lines were measured by thiobarbituric acid (TBA) assay (Ohkawa et al., 1979). Briefly, the reaction mixture contained 1.5 ml acetic acid (20%), 250 µl of fly head homogenate, 1.5 ml of TBA (0.8% w/v) and 200 µl sodium lauryl sulphate. The mixture was incubated in a boiling water bath for 45 min and extracted into 3 ml of 1-butanol. Absorbance was measured at 535 nm and quantified as malondialdehyde equivalents normalized to the total protein content.

### Immunohistochemistry

Immunohistochemistry was performed to detect mutant Htt on the paraffin-embedded sections of fly heads for all experimental genotypes as described previously (Rai et al., 2018). Briefly, sections were deparaffinized, hydrated and processed for antigen retrieval, and then incubated in primary antibodies at 4°C overnight. Post incubation, sections were washed and incubated with corresponding horseradish peroxidase (HRP)-tagged secondary antibodies, and signals were visualized using the DAB kit (Genei Laboratories Pvt. Ltd). Images were quantified using ImageJ software by setting a threshold for MW8-positive particles, and by measuring the percentage area and average size above the threshold.

### Immunoblotting

For GS western blotting, 20–30 fly heads for each genotype were homogenized in 1× RIPA buffer supplemented with the 1× protease and 1× phosphatase inhibitor cocktail. An equal amount of protein was loaded, separated using 10% SDS-polyacrylamide gel electrophoresis and transferred onto a nitrocellulose membrane. The membrane was then blocked in 5% non-fat dry milk blocking buffer and incubated in primary and secondary antibodies according to the manufacturer' protocol. Immunoreactive bands were detected using the chemiluminescent detection kit SuperSignal West Pico PLUS Chemiluminescent Substrate (catalog no.: 34580, Thermo Fisher Scientific), and analyzed using the ChemiDoc XRS+ gel imaging system (Bio-Rad Laboratories India Pvt. Ltd) (Sinha et al., 2021).

### Soluble-insoluble fractionation

For the fractionation of Htt aggregates in fly heads, 40–50 flies per genotype were homogenized in 100 µl of 1% Triton X-100 supplemented with protease and phosphatase inhibitor cocktail, incubated on ice for 20 min, and centrifuged at 14,000 ***g***. Supernatants were collected and designated as detergent-soluble fractions. Pellets were washed with the lysis buffer, suspended in 1× Laemmli buffer and designated as detergent-insoluble fractions. An equal amount of total tissue protein was analyzed using 8% SDS-PAGE and processed for standard immunoblotting protocol (Steffan et al., 2001). Ubiquitin soluble-insoluble fractionations were performed as described previously (Kinghorn et al., 2016).

### LysoTracker staining

Brains of adult flies of the experimental genotypes were dissected in PBS and transferred to a solution of PBS containing 1 μm LysoTracker RED and 1 μm DAPI for 5 min. Post incubation, the fly brains were mounted on slides and lysosomes imaged using a Nikon two-photon microscope (Nikon A1RMP+) with a 16× objective. Images were quantified using the ImageJ software by setting a threshold for LysoTracker RED, and measuring the area and intensity above the threshold (Kinghorn et al., 2016).

### Protein estimation

The protein content for all biochemical assays, including activity of GS, estimated levels of glycogen, measurement of reactive oxygen species (ROS) and lipid peroxidation, was determined by using the bicinchoninic acid (BCA) method as described by Smith et al. (1985). To prepare lysates for specific assays, a small portion of a sample was isolated and utilized to determine its total protein content. This was done by measuring the absorbance of the BCA−Cu^+^ complex at the wavelength of 562 nm using spectrophotometry. The protein content in mg/ml was calculated by plotting the absorbance values against standards for bovine serum albumin.

### Statistical analysis

Statistical data analyses were carried out using the GraphPad Prism software (Version 7, GraphPad Software Inc., California). For survival assay, Kaplan–Meier survivorship curves and log-rank (also known as Mantel-Cox) test values were used to calculate the significance. Data for all parameters were either subjected to two-tailed unpaired *t*-test or factorial analysis of variance (ANOVA) as indicated. Multiple comparisons were carried out using Tukey's post-hoc test. A *P* <0.05 value was considered to be significant.

## Supplementary Material

10.1242/dmm.050238_sup1 Supplementary information

## First Person

This article has an associated First Person interview with the first author of the paper.
