# Supplementary material for: Increase in brain glycogen levels ameliorates Huntington's disease phenotype and rescues neurodegeneration in Drosophila
Source: Dis Model Mech. 2023 Oct 19;16(10):dmm050238. doi: 10.1242/dmm.050238 (PMC10602008; doi:10.1242/dmm.050238)
Supplement: Supplementary information [file dmm-16-050238-s1.pdf]

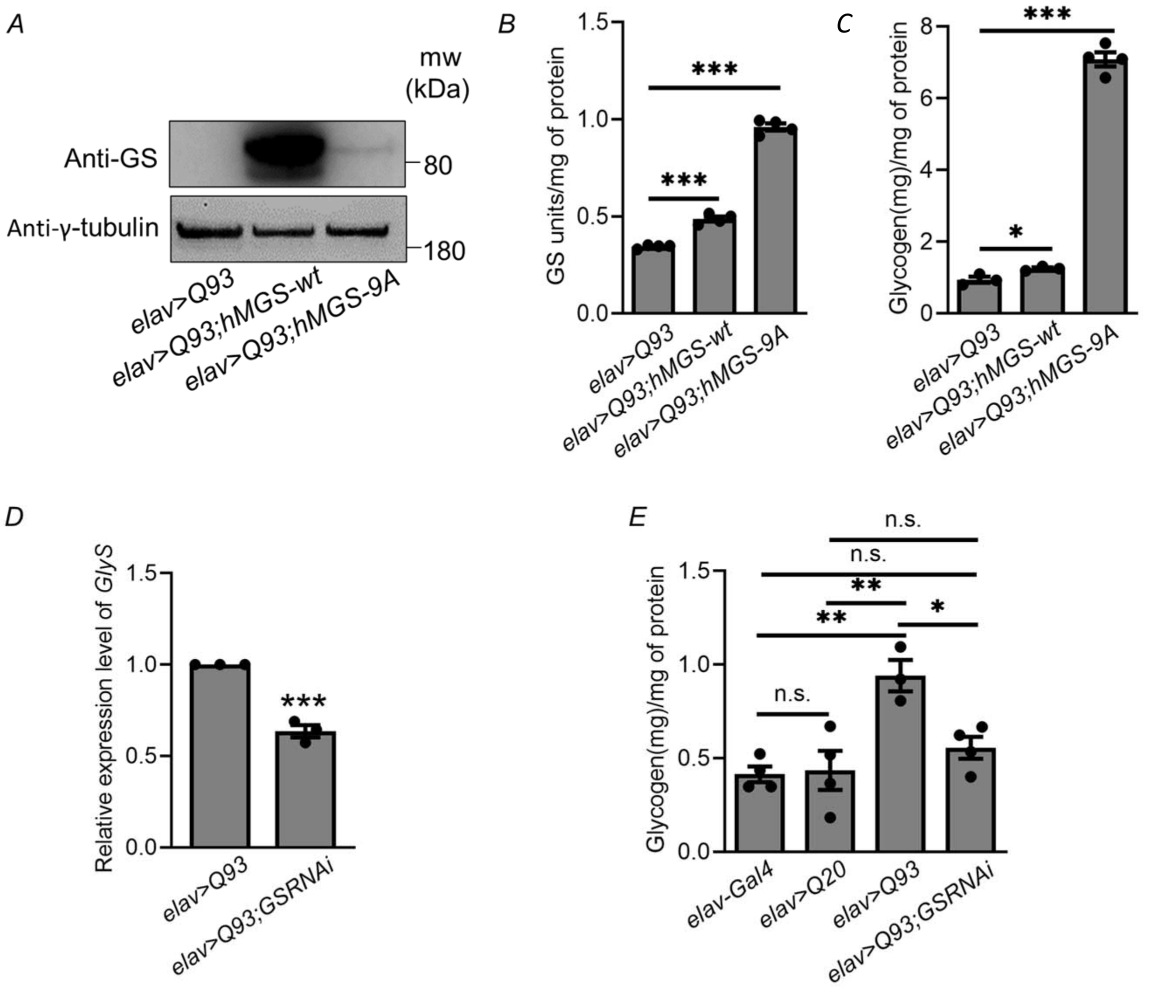

Fig. S1. Validation of the transgenic lines used in the study: ***(A)*** Representative immunoblots for the head extracts of one-week-old flies showing the immunoreactivity in the *elav>Q93;hMGS-wt* and *elav>Q93;hMGS-9A* genotypes for the mouse/human-specific GS antibody. *elav>Q93* served as the control. γ-tubulin served as the loading control. (***B, C***) Bar diagram showing the GS activity ***(B)*** and absolute glycogen levels ***(C)*** in the brain tissues of one-week-old *elav>Q93, elav>Q93;hMGS-wt,* and *elav>Q93;hMGS-9A* flies. The values for both are normalized to the total protein content. ***(D)*** Bar diagram showing the expression level (transcript) of GS (*GlyS*) in one-week-old fly heads of *elav>Q93* and *elav>Q93;GSRNAi* flies. The expression values for GS transcript were normalized to that of the housekeeping gene *Rpl32*. ***(E)*** Bar diagram showing the absolute glycogen levels in one-week-old fly heads of *elav-Gal4*, *elav>Q20* (controls), the HD fly (*elav>Q93*) and the HD fly with GS knockdown (*elav>Q93;GSRNAi*). The values are normalized to the total protein content. Each bar represents the mean ± SE (*p<0.05,** p<0.01*** p<0.001; N=3-4). GS, glycogen synthase.

Disease Models & Mechanisms • Supplementary information

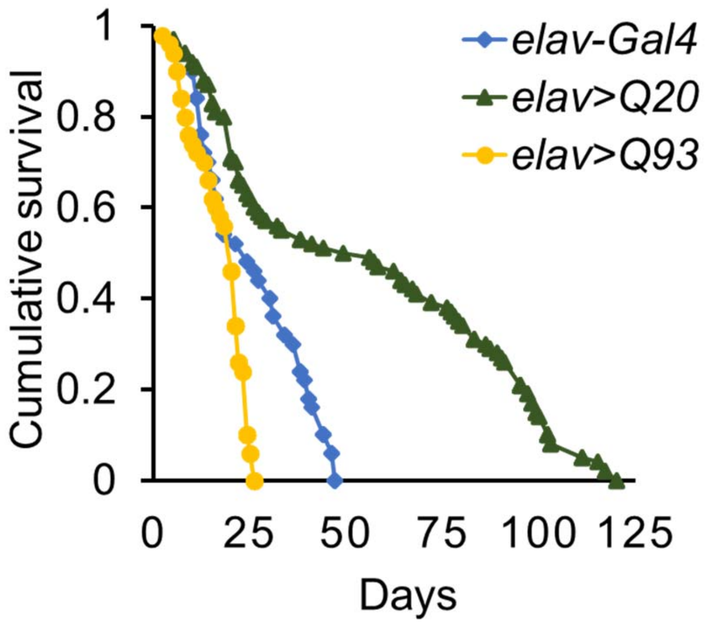

Fig. S2. Survival in the Huntington's disease fly model: Kaplan-Meier survivorship graphs showing cumulative survival in days for *elav-Gal4* and *elav>Q20* (controls) and flies with a pan-neuronal overexpression of mutant huntingtin (*elav>Q93*). Data were analyzed using the Log-rank test using Mantel-Cox ($\chi 2$ = 152.83) and the p-value (0.001).

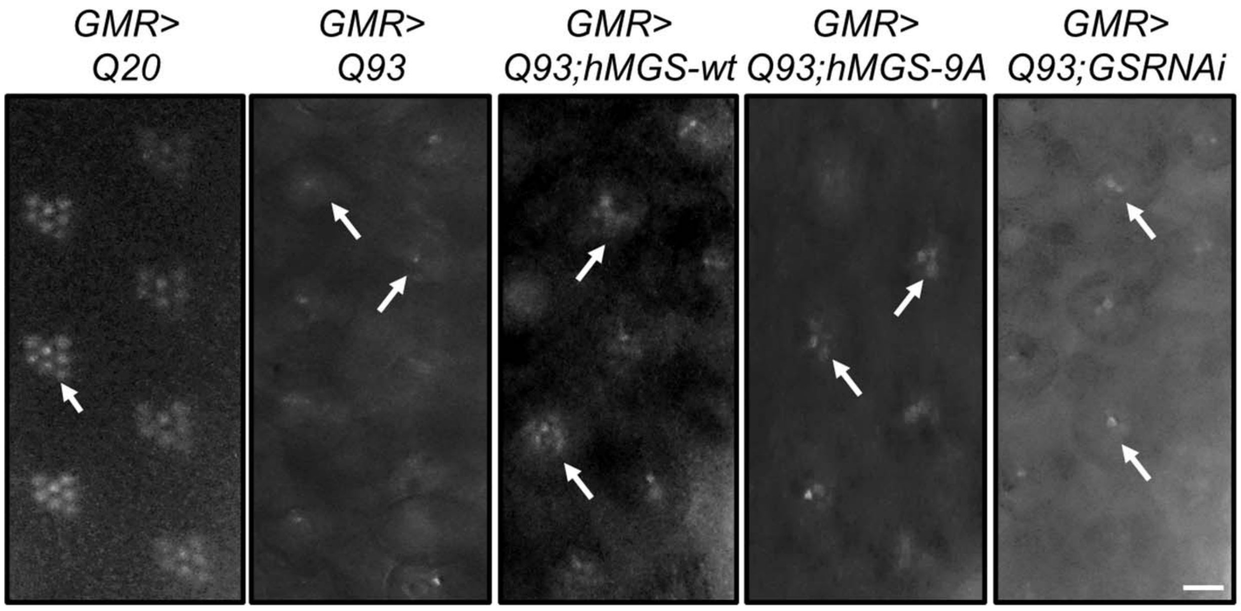

Fig. S3. Enhanced glycogen synthesis alleviates rhabdomere degeneration in the Huntington's disease fly: Pseudopupil analysis of one-week-old *GMR>Q20* (control), *GMR>Q93, GMR>Q93;hMGS-wt, GMR>Q93;hMGS-9A* and *GMR>Q93;GSRNAi* flies. Arrows in represent the distinct rhabdomeres observed in the experimental genotypes [Scale bar, 50µm; N=3]. GS, glycogen synthase

Disease Models & Mechanisms • Supplementary information

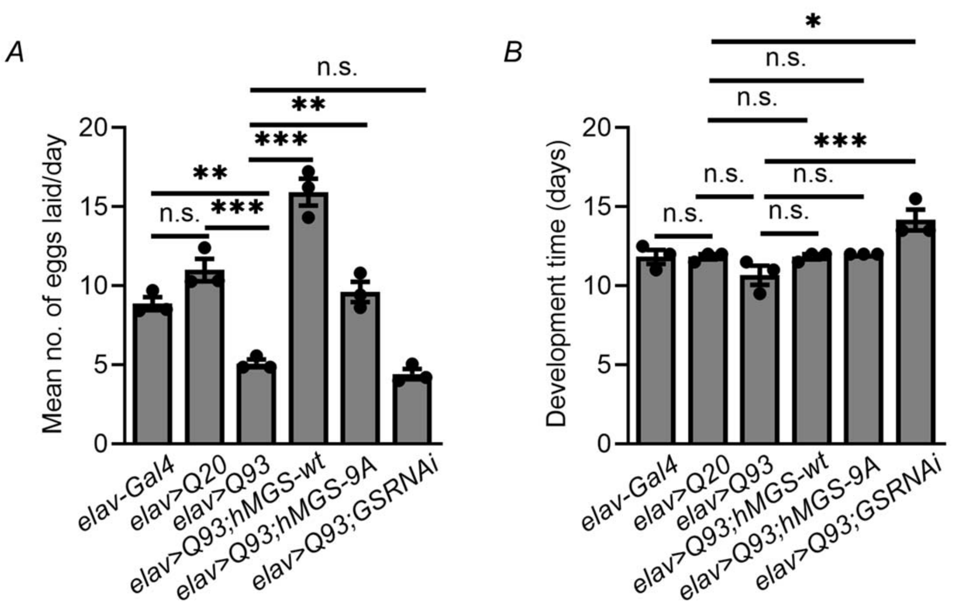

Fig. S4. GS overexpression improves fertility in the Huntington's disease fly model: ***(A)*** Average total daily fecundity/fly and (***B***) overall development time (days) in *elav-Gal4* (control), *elav>Q93* (HD fly), *elav>Q93;hMGS-wt* and *elav>Q93;hMGS-9A* (HD fly with pan-neuronal overexpression of hMGS) and with a knockdown of GS (*elav>Q93;GSRNAi*). Each value represents the mean ± SE (*p<0.05, **p<0.01, ***p<0.001, n.s., not significant; N=50). GS, glycogen synthase

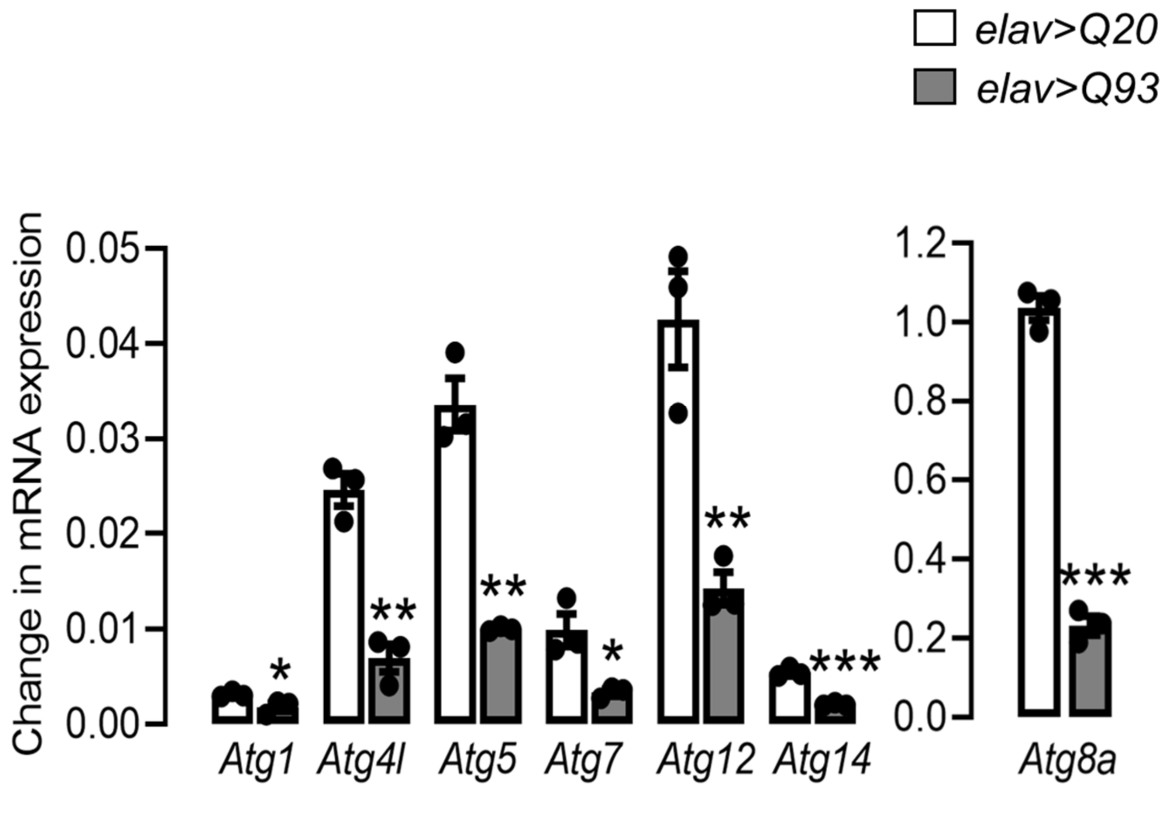

Fig. S5. Levels of autophagy-related gene (ATGs) transcripts in the brain of Huntington's disease fly: Bar diagram showing the expression level (transcript) of ATGs in one-week-old fly heads of *elav>Q20* (control) and *elav>Q93* flies. The expression values for the GS transcript were normalized to that of the housekeeping gene *Rpl32*. Each bar represents the mean ± SE (*p<0.05, **p<0.01, ***p<0.001; N=3). GS, glycogen synthase

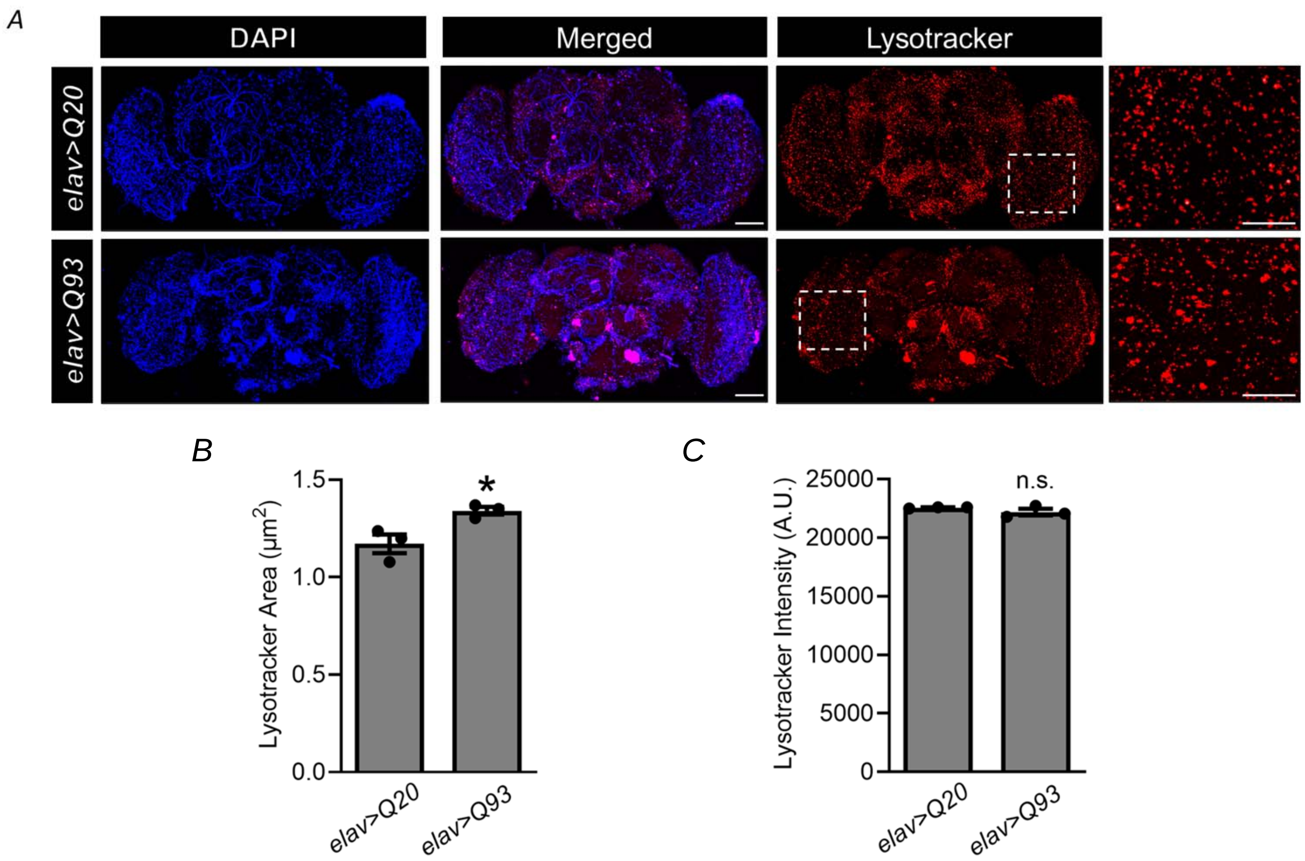

Fig. S6. Lysosome architecture and intensity in the Huntington's disease fly *(A)* Representative fluorescence images for the lysotracker staining (red) marking the lysosomes in the one-week-old fly brain of *elav>Q20* (control) and *elav>Q93* flies. Nuclei were stained with DAPI (blue). Scale bar, 60µm. Bar diagram representing ***(B)*** the area (μm^2) and ***(C)*** intensity quantification of the lysosome puncta in the fly brain of *elav>Q20* (control) and *elav>Q93* flies. Each bar represents the mean value ± SE. ($^*p<0.05$, n.s., not significant, N=3-4).

Table S1. Sequence of gene-specific primers used for the expression studies.

**Gene name**	**Symbol**	**Forward primer (5'to 3')**	**Reverse primer (5'to 3')**
Ribosomal protein L32	*Rpl32*	ATCGGTTACGGATCGAACAA	GACAATCTCCTTGCGCTTCT
Glycogen synthase	*GlyS*	GTTATTCGTTTTGTTTCGTGTGGC	CGAGCGCAATGAGTTGACAG
Autophagy-related 8a	*Atg8a*	ATTCCACCAACATCGGCTAC	GCCATGCCGTAAACATTCTC
Autophagy-related 7	*Atg7*	GCGACGATGGCCAAGAAATC	CTCTGTGCATAGTAGGCGGG
Autophagy-related 12	*Atg12*	CTGGCAATGTGCCCATCATCA	TAGCCCCACGCCTGATTCTT
Autophagy-related 4b	*Atg4b*	CCCATCAAACCGAGCAAACAGA	TTATGTGGTGGTGGCGGCAA
Autophagy-related 14	*Atg14*	CCATCTGGACGTGAACAATG	GCAGAGAGTTTTCGTCCTCT
Autophagy-related 5	*Atg5*	GCACGCACGGCATTGATCTACA	GCCCTGGGATTTGCTGGAAT
Autophagy-related 1	*Atg1*	GAGTATTGCAATGGCGGCGACT	CAGGAATCGCGCAAACCCAA

Disease Models & Mechanisms • Supplementary information
